# Simultaneous Uniportal video-assisted thoracic surgery of bilateral pulmonary nodules

**DOI:** 10.1186/s13019-021-01423-z

**Published:** 2021-03-22

**Authors:** Shengcheng Lin, Chenglin Yang, Xiaotong Guo, Yafei Xu, Lixu Wang, Zhe Wang, Xin Yu, Chunguang Wang, Zhentao Yu

**Affiliations:** 1 Department of Thoracic Surgery, National Cancer Center/National Clinical Research Center for Cancer/Cancer Hospital and Shenzhen Hospital, Chinese Academy of Medical Sciences and Peking Union Medical College, Shenzhen, 518116 China; 2 Department of Anesthesiology, Shunde Hospital of Southern Medical University (The First People’s Hospital of Shunde Foshan), Foshan, China

**Keywords:** Simultaneous, Bilateral, Pulmonary nodules, Uniportal video-assisted thoracic surgery, Postoperative complications

## Abstract

**Background:**

Surgical resection is an appropriate treatment option for synchronous bilateral pulmonary nodules with ground-glass opacities. The applicability of simultaneous uniportal video-assisted thoracic surgery is not fully understood. We evaluated the feasibility and safety of performing such surgeries at our hospital.

**Methods:**

Clinical data of 35 patients who underwent simultaneous bilateral pulmonary resection with uniportal video-assisted thoracic surgery at our hospital were reviewed retrospectively.

**Results:**

Simultaneous bilateral pulmonary resection with uniportal video-assisted thoracic surgery was performed for 35 patients (15 men, 20 women); 97 nodules were operated on, and the average nodule diameter was 11.4 mm (range, 1–38 mm). Computerized tomography showed that most nodules had ground-glass opacity (52/97, 53.6%); solid nodules (24/97, 24.7%) and nodules with mixed ground-glass opacity (21/97, 21.7%) were noted. Surgical resection included lobar-sublobar resection (11/35, 31.4%) and sublobar-sublobar resection (24/35, 68.6%). Wound infection and postoperative 30-day mortality were not observed. Pneumonia was the major postoperative complication, with a higher incidence in the lobar-sublobar group (6/10, 60%) than in the sublobar-sublobar group (4/25, 16%; *P* = 0.016). Pneumonia did not correlate with operative time (mean, 262.3 ± 108.1 vs. 261.9 ± 87.5 min, *P* = 0.991), duration of chest drainage (mean, 7.0 ± 4.0 vs 5.4 ± 2.1 days, *P* = 0.124), and postoperative hospital stay (mean, 10.2 ± 3.6 vs 10.2 ± 6.4 days, *P* = 0.978). The mean follow-up time was 8 (range, 3–22) months. Recurrence of primary lung cancer or mortality was not noted at the final follow-up.

**Conclusions:**

Simultaneous bilateral pulmonary resection with uniportal video-assisted thoracic surgery is feasible and safe for appropriate patients. Simultaneous lobar-sublobar pulmonary resection for bilateral nodules can increase the risk of developing pneumonia.

## Background

Due to the popularization of thin-section computed tomography (CT) and the increasing awareness of people’s health examinations, an increasing number of synchronous bilateral pulmonary nodules (SMBPNs) have been found, mostly ground-glass opacities (GGO) [1, 2]. It is necessary to distinguish the nature of multiple pulmonary nodules—benign or malignant—and whether it is a multifocal primary lung cancer or intrapulmonary metastasis. Therefore, it can be challenging to choose the appropriate treatment option.

Surgical resection has become the main treatment for SMBPNs highly suspected to be synchronous multiple primary lung cancers (SMPLCs). A 35–92% 3-year survival rate of SMPLCs has been reported in the literature [3, 4]. Simultaneous bilateral pulmonary resections have been considered to have high invasiveness and mortality, and their safety is still debated [3, 5]. Since Japanese researchers initially reported simultaneous bilateral pulmonary resections 10 years ago, there have been several studies of simultaneous operations for SMPLCs [2, 6]. A few studies have reported the efficacy of uniportal video-assisted thoracic surgery (VATS) in the treatment of SMPLCs. Here, we summarize our hospital’s experience with the feasibility and safety of simultaneous uniportal video-assisted thoracic surgery in the treatment of SMPLCs within the last 2 years, particularly evaluating the associated postoperative complications.

## Methods

### Patients and clinical features

This study was approved by the Cancer Hospital and Shenzhen Hospital, Chinese Academy of Medical Sciences and Peking Union Medical College Institutional Review Board of Clinical Research (NO.: 2020–173). The study conformed to the provisions of the Declaration of Helsinki. Due to the retrospective study design, the informed consent requirement was waived. Clinical data of 35 patients who underwent simultaneous bilateral pulmonary resection by uniportal VATS at our hospital from April 2018 to January 2020 were reviewed retrospectively.

An institutional oncologic multidisciplinary meeting (attended by experts from the surgery, radiation oncology, and medical oncology departments) was held to evaluate preoperative treatment strategies. All patients in this study were cN0 without distant metastasis, and no patients demonstrated pathologic mediastinal lymph node.

Imaging characteristics and the consolidation/tumor ratio (CTR) of tumors were evaluated using thin-section CT. In our research, the computed tomography (CT) features of tumors were divided into the following three subgroups according to the imaging characteristics and the CTR of the tumors: pure GGO (pGGO) group, CTR = 0; mixed GGO (mGGO) group, CTR > 0 and CTR < 1; solid pulmonary nodules (sPNs) group, CTR = 1 [1].

The TNM staging depended on the most advanced stage of all nodules in each primary lung cancer case. All postoperative histopathologic-proven adenocarcinomas were reclassified based on the new classification proposed by the International Association for the Study of Lung Cancer [7]. The pathologic criteria for diagnosis of SMPLCs in our hospital were based on the Martini-Melamed criteria [8]/Antakli criteria [9].

Diagnosis of postoperative pulmonary complications was performed based on EPCO-recommended definitions [10], where the definition of pneumonia includes at least one of the following items: infiltration, consolidation, and cavitation; plus at least one of the following: fever > 38 °C without other cause, white cell count < 4 or > 12 × 10^9^/L, age > 70 years with altered mental status without other cause; plus at least two of the following: new purulent/changed sputum, increased secretions/suctioning, new/worse cough/dyspnea/tachypnea, rales/bronchial breath sounds, and worsening gas exchange.

### Surgical procedure

Simultaneous bilateral pulmonary resection by uniportal VATS was performed by the same team of surgeons and anesthetists using double-lumen endotracheal intubation. Pulmonary resection procedures were selected based on the size, location, and TSCT characteristics of tumors, as well as the performance status and pulmonary function testing. The sPNs or mGGO (CTR > 0.5) were usually resected by lobectomy, whereas pGGO or mGGO (CTR < 0.5) were usually resected by segmentectomy or wedge resection. For patients with insufficient pulmonary reserve or poor performance, segmentectomy or wedge resection was selected instead of lobectomy. The priority for surgical resection was given to the side with a smaller resection range, and the right side was chosen in cases where both sides were similar.

### Follow-up

All patients were followed up with chest CT and serum tumor markers (pro-gastrin-releasing peptide, carcinoembryonic antigen, cytokeratin 19 fragments, neuron-specific enolase, carbohydrate antigen, and alpha-fetoprotein) every 3 months and with cranial MRI and abdominal CT (including adrenals) every half year, and whenever necessary with positive symptoms at our clinic.

### Statistical analysis

Continuous variables are presented as the mean and standard deviation and median and range; categorical variables are presented as numbers and percentages. To compare the postoperative course according to perioperative characteristics, Mann-Whitney test was used for quantitative variables, and the χ^2^ test or Fisher test was used for qualitative variables. SPSS, version 20 for Windows (IBM Corp, Armonk, NY) was used for statistical analysis. A *P* value < 0.05 was considered statistically significant.

## Results

### Clinical characteristics

Simultaneous bilateral pulmonary resection by uniportal VATS was performed in 35 patients (2 of the 37 patients did not have complete follow-up data). Our patients’ cohort comprised 15 men and 20 women, with mean age 60.2 ± 8.9 [range, 42–75] years. Three male patients had a history of smoking, and eight patients had comorbidities, including diabetes mellitus (*n* = 3), hypertension (*n* = 1), tuberculosis (*n* = 2), coronary heart disease (*n* = 1), and diabetes mellitus combined with hypertension (*n* = 1). The mean preoperative-forced expiratory volume in 1 s (FEV 1) was 2486.9 mL (range, 1680–4560 mL), and the FEV 1 mean forced vital capacity (FVC) was 91.0% (range, 75.7–99.9%).

Ninety-seven nodules were operated on, and the average nodule diameter was 11.4 mm (range, 1–38 mm). TS CT revealed that most nodules were pGGO (52/97, 53.6%), followed by sPNs (24/97, 24.7%) and mGGO (21/97, 21.7%) nodules. In all cases, the performance status values ranged from 0 to 1 (Table 1).

**Table 1:** Clinical characteristics (*N* = 35)

Variable	Mean ± SD (range) or No. (%)
Age (year)	60.2 ± 8.9 (42–75)
Sex	
Male	15 (42.9)
Female	20 (57.1)
Smoking history	
Yes	3 (8.6)
No	32 (91.4)
Comorbidity	
Diabetes mellitus	4 (11.4)
Hypertension	2 (5.7)
Tuberculosis	2 (5.7)
Coronary heart disease	1 (2.8)
Pulmonary function	
Forced expiratory volume in 1 s	2486.9 ± 644.3 (1680–4560)
FEV1 mean forced vital capacity	91.0 ± 7.4 (74.5–99.9)
Number of tumors	
> 2	14 (40)
< 2	21 (60)
CF	
pGGO	52 (53.6)
mGGO	21 (21.7)
sPNs	24 (24.7)

*FEV1* preoperative-forced expiratory volume in 1 s, *CF* classification, *pGGO* pure ground-glass opacity, *mGGO* mixed GGO, *sPNs* solid pulmonary nodules

### Surgery and pathology

All patients underwent bilateral 3–4 cm incisions in the fourth (for upper lobar/sublobar resection in right lung) or fifth (for other pulmonary resection) interspace between the posterior and middle axillary line. A 20F silicone chest tube was placed in the same incision through a muscle tunnel. Lobar-sublobar (L-SL) resection (11/35, 31.4%) included lobectomy and contralateral segmentectomy (7/35, 20.0%), lobectomy and contralateral wedge resection (3/35, 8.6%), and lobectomy and contralateral multiple sublobar resection (segmentectomy and wedge resection) (1/35, 2.8%). Sublobar-sublobar (SL-SL) resection (24/35, 68.6%) included segmentectomy and contralateral segmentectomy (14/35, 40.0%), and multiple wedge resection (10/35, 28.6%). The mean duration of surgery and blood loss was 262.7 ± 90.4 (range, 116–478) min and 104 ± 110.3 mL (range, 20–600 mL), respectively.

Postoperative histopathologic diagnoses were made based on the Martini-Melamed criteria [8] and Antakli criteria [9]. The selected patients had SMPLCs (23/35, 65.7%), single primary lung cancer (8/35, 22.9%), bilateral atypical adenomatous hyperplasia (AAH) (1/35, 2.8%), and bilateral benign nodules (3/35, 8.6%). All patients with primary lung cancer were classified as IA1 (10/35, 28.6%), IA2 (9/35, 25.7%), IA3 (8/35, 22.9%), IB (1/35, 2.8%), IIB (1/35, 2.8%), and IIIA (1/35, 2.8%). The most advanced stage of one patient with lung cancer was adenocarcinoma in situ (AIS). One patient with a p-stage IIIA disease had T3 N1 cancer.

All nodules included invasive adenocarcinoma (53/97, 54.7%), minimally invasive adenocarcinoma (MIA) (8/97, 8.2%), AIS (8/97, 8.2%), AAH (8/97, 8.2%), and benign nodules (20/97, 20.7%). Postoperative histopathologic examination revealed that 71.1% (69/97) of nodules were lung adenocarcinoma, with 39.2% of those nodules being pGGO (38/97), followed by 17.5% mGGO (17/97), and 14.4% sPNs (14/97) (Table 2).

**Table 2:** Surgical and pathologic characteristics (*N* = 35)

**Variable**			**No. (%)**			
Resection (right/left)						
L/S-W			1 (2.8)			
L/S			7 (20.0)			
L/W			3 (8.6)			
S/S			14 (40.0)			
W/W			10 (28.6)			
Postoperative histopathologic						
SMPLCs			23 (65.7)			
Single primary lung cancer			8 (22.9)			
Bilateral AAH			1 (2.8)			
Bilateral benign nodules			3 (8.6)			
P-stage						
IA1			10 (28.6)			
IA2			9 (25.7)			
IA3			8 (22.9)			
IB			1 (2.8)			
IIB			1 (2.8)			
IIIA			1 (2.8)			
Others			5 (14.4)			
Pathology n/%						
	IA^a^	MIA	AIS	BN	AAH	Total
CF n/%						
pGGO	25 (25.8)	7 (7.2)	6 (6.2)	8 (8.2)	6 (6.2)	52 (53.6)
mGGO	15 (15.5)	1 (1.0)	1 (1.0)	3 (3.2)	1 (1.0)	21 (21.7)
sPNs	13 (13.4)	0	1 (1.0)	9 (9.5)	1 (1.0)	24 (24.9)
Total	53 (54.7)	8 (8.2)	8 (8.2)	20 (20.7)	8 (8.2)	97 (100)

*L* lobectomy, *S* segmentectomy, *W* wedge resection, *SMPLC* synchronous multiple primary lung cancers, *AAH* atypical adenomatous hyperplasia; Others, including adenocarcinoma in situ, bilateral AAH and other benign diseases. *IA*^a^ invasive adenocarcinoma, *MIA* minimally invasive adenocarcinoma, *AIS* adenocarcinoma in situ, *BN* benign nodules, *CF* classification, *pGGO* pure ground-glass opacity, *mGGO* mixed GGO, *sPNs* solid pulmonary nodules

### Postoperative course

No wound infection and postoperative 30-day mortality occurred. The average time interval for chest tube placement and postoperative hospital stay was 5.8 ± 2.7 (range, 3–14) days and 10.3 ± 5.7 (range, 5–35) days, and the median was 5 days and 8 days, respectively. Postoperative complications [10] included pneumonia (*n* = 6), air leak (*n* = 1), atrial fibrillation (*n* = 2), and pneumonia combined with air leak (*n* = 1). We analyzed several preoperative variables in accordance with the operative time, days of drainage, postoperative hospital stay, and rate of complications (Table 3). Pneumonia was the main postoperative complication. The incidence of pneumonia in the L-SL group (6/10, 60%) was higher than in the SL-SL group (4/25, 16%; *P* = 0.016); however, this did not correlate with the operative time (mean, 262.3 ± 108.1 vs. 261.9 ± 87.5 min, *P* = 0.991), the time of chest drains (mean, 7.0 ± 4.1 vs. 5.4 ± 2.1 days, *P* = 0.124), and postoperative hospital stay (mean, 10.2 ± 3.6 vs. 10.2 ± 6.4 days, *P* = 0.978) (Table 3).

**Table 3:** Comparison of several variables with clinical outcomes

Variable	Operative Time Mean ± SD (range)	Chest Tubes Mean ± SD (range)	Hospital Stay Mean ± SD (range)	Complications n (%)
Age				
> 60 y	274.4 ± 93.4	5.6 ± 2.4	10.5 ± 6.9	7 (38.9)
≤ 60 y	247 ± 89.2	5.6 ± 2.7	9.8 ± 4.2	3 (17.7)
	*P* = 0.387	*P* = 1	*P* = 0.722	*P* = 0.264
Lobectomy				
Yes	262.3 ± 108.1	7.0 ± 4.1	10.2 ± 3.6	6 (60.0)
No	261.9 ± 87.5	5.4 ± 2.1	10.2 ± 6.4	4 (16.0)
	*P* = 0.991	*P* = 0.124	*P* = 0.978	*P* = 0.016
Systematic lymphadenectomy				
Yes	291.3 ± 86.6	6.1 ± 3.1	11.0 ± 6.44	8 (32.0)
No	191.8 ± 62.4	5.1 ± 1.5	8.3 ± 2.9	2 (20.0)
	*P* = 0.002	*P* = 0.346	*P* = 0.222	*P* = 0.686
FEV1/FVC				
> 90%	238.9 ± 92.5	6.5 ± 2.9	11.3 ± 6.8	7 (36.8)
≤ 90%	288.3 ± 86.2	5 ± 2.4	9.4 ± 4.1	3 (18.8)
	*P* = 0.119	*P* = 0.111	*P* = 0.325	*P* = 0.258

*SD* standard deviation, *FEV1* preoperative-forced expiratory volume in 1 s, *FVC* FEV 1 mean forced vital capacity

### Follow-up and survival

The mean follow-up time was 8 (range, 3–22) months. There was no recurrence of primary lung cancer or death at final follow-up.

## Discussion

In 1898, a patient with multiple primary cancers was reported by Billroth [11]; however, the concept of multiple primary lung cancers (MPLCs) was first described by Beyreuther in 1924 [11]. Martini and Melamed [8] published the first diagnostic criteria in 1975, and Antakli et al. [9] supplemented the diagnostic criteria for second primary lung cancer in 1995 and incorporated elements of the new international multidisciplinary lung adenocarcinoma classification [12]. According to these criteria, MPLCs include two subsets frequently referred to as SMPLCs and metachronous multiple primary lung cancers (MMPLCs).

Due to diagnostic radiographic techniques and the introduction of helical CT in the screening of lung cancers, an increasing number of SMBPNs with GGO components have been identified, and many have proven to be SMPLCs. The incidence of SMPLCs in the reported literature varies from 0.7 to 6.1% of all lung cancer cases [3, 13]. According to our data, postoperative histopathology revealed that 65.7% of the cases had SMPLCs (23/35), 22.9% had single primary lung cancer (8/35), 2.86% had bilateral AAH (1/35), and 8.6% had bilateral benign nodules (3/35). The high incidence of SMPLCs is due to the preoperative multidisciplinary meeting of the institutional oncology team and the careful selection of therapeutic strategies, especially for SMBPNs containing GGO components. Hence, 71.1% (69/97) of nodules were confirmed to be lung adenocarcinoma, with 56.7% of nodules (55/97) having GGO components, including 39.2% with pGGO nodules (38/97) and 17.5% with mGGO nodules (17/97); additionally, solid nodules (14/97) did not appear to be numerous.

Surgical resection with curative-intent for bilateral nodules had a better survival rate than ipsilateral operation and contralateral combination therapy as well as non-surgical therapy [14]; especially for the node-negative subgroup [15], this was an appropriate treatment option. In the past, lobectomy was considered a standard surgical treatment for patients with stage I NSCLC [16]. Staged bilateral lobectomy with intervals of 4 to 6 weeks was more feasible in patients with bilateral lung cancers than simultaneous bilateral lobectomy [17]. However, in clinical stage IA lung adenocarcinoma with GGO component (CTR < 0.5), sublobar resection has been shown to be equivalent to lobectomy with regards to survival [18, 19], even for solid nodules [19]. Compared with staged bilateral pulmonary resection, simultaneous bilateral pulmonary resection (L-SL/SL-SL) is considered an effective strategy with shorter postoperative in-hospital stay, similar postoperative complications and duration of chest drain usage [6]. Compared with traditional multi-portal VATS, uniportal VATS has become a safe and feasible technique at our center, with similar duration of postoperative hospital stay and decreased postoperative thorax drainage [20]. Yao et al. [2] reported that the SL-SL group required less hospitalization after surgery than the L-L/SL group. However, in the present study, there was no significant change in hospital stay between the L-SL group and the SL-SL group.

The optimal surgical methods and suitable timing for synchronous bilateral lung cancer are still debated. Tanvetyanon et al. [21] analyzed 467 patients with multi-lobe synchronous multiple lung cancers. The results revealed that the histology of adenocarcinoma could independently predict the survival rate in multivariate analysis combining histology, sex, age, maximum T-size, highest N-stage, and laterality. They also highlighted that several N0 and T sized ≤3 cm, patients under 70 years of age, and female sex were favorable survival predictors. This research analyzed survival predictors for surgeons to select appropriate patients; unfortunately, it did not mention the surgical methods—whether single-stage or two-stage. Mun and Kohno [6] suggested a performance status of 3 or higher, when the predicted postoperative FEV 1 was < 800 mL, and bilateral lobectomy recommended staged pulmonary resections. Our research with 35 patients was selected on the premise that patients had good performance status (0–1), an FEV 1/FVC greater than 75%, and were cN0. To the best our knowledge, this is the largest study of surgical outcomes in patients with SMPLCs who underwent simultaneous bilateral pulmonary resection. There was no postoperative 30-day mortality, and the postoperative course was uneventful in 71.4% of cases. In the current study, the rate of postoperative complications was 28.6% (10/35), including pneumonia (*n* = 6), air leak (*n* = 1), atrial fibrillation (*n* = 2), pneumonia combined with air leak (*n* = 1), and respiratory and cardiac morbidities after the operation had been reported [10]. Of those with postoperative complications, the incidence of pneumonia was higher in the L-SL group than in the SL-SL group with a similar operative time. In the past few years, the duration of surgical resection (> 2 h) has presented an increased risk of pulmonary complications [10]; however, in our study—with a similar operative time and higher pneumonia morbidity—simultaneous bilateral SL-SL resection was the preferred therapeutic option compared to simultaneous bilateral L-SL resection, even if both are feasible and safe.

The results of this study indicate that simultaneous bilateral pulmonary resection by uniportal VATS may be a safe and feasible technique for patients with performance status (0–1), FEV 1/FVC more than 75%, and who are cN0. Given these conditions, for SMBPNs with GGO-dominant (CTR < 0.5), it is preferable to opt for simultaneous SL-SL pulmonary resection. For SMBPNs with GGO component (CTR > 0.5) contralateral GGO component (CTR < 0.5), it is preferable to opt for simultaneous L-SL pulmonary resection for selected patients. Additionally, for those patients with poor performance status and pulmonary function testing, a staged resection along with a bilateral lobectomy is preferred.

Finally, our research had several limitations. First, the selection of patients was biased because this is a single-center retrospective study. Second, the sample size was small, as only 35 patients underwent the evaluation. Finally, the follow-up time was short, and the evaluation of long-term survival is lacking. Therefore, multi-center design research with a larger sample size and a longer follow-up time is required to verify our conclusions.

## Conclusions

Simultaneous bilateral pulmonary resections by uniportal VATS is a feasible and safe procedure in appropriate patients. However, simultaneous L-SL pulmonary resections for bilateral nodules can increase the risk of developing pneumonia.

## Data Availability

Not applicable.

## Abbreviations

CT: Computed tomography
SMBPNs: Synchronous bilateral pulmonary nodules
GGO: Ground-glass opacities
SMPLCs: Synchronous multiple primary lung cancers
VATS: Video-assisted thoracic surgery
CTR: Consolidation/tumor ratio
pGGO: Pure ground-glass opacities
mGGO: Mixed ground-glass opacities
sPNs: Solid pulmonary nodules
FEV 1: Forced expiratory volume in 1 s
FVC: Forced vital capacity
L-SL: Lobar-sublobar
SL-SL: Sublobar-sublobar
AAH: Atypical adenomatous hyperplasia
AIS: Adenocarcinoma in situ
MIA: Minimally invasive adenocarcinoma
CF: Classification
MPLCs: Multiple primary lung cancers
MMPLCs: Metachronous multiple primary lung cancers

## References

[1] Zhang Y, Fu F, Chen H (2020). Management of ground-glass opacities in the lung cancer spectrum. Ann Thorac Surg.

[2] Yao F, Yang H, Zhao H (2016). Single-stage bilateral pulmonary resections by video-assisted thoracic surgery for multiple small nodules. J Thorac Dis.

[3] Nakata M, Sawada S, Yamashita M, Saeki H, Kurita A, Takashima S, Tanemoto K (2004). Surgical treatments for multiple primary adenocarcinoma of the lung. Ann Thorac Surg.

[4] Shah AA, Barfield ME, Kelsey CR, Onaitis MW, Tong B, Harpole D, D'Amico TA, Berry MF (2012). Outcomes after surgical management of synchronous bilateral primary lung cancers. Ann Thorac Surg.

[5] Iino K, Oda M, Tsunezuka Y, Ota Y, Watanabe G, Minato H (2002). Treatment for bilateral multiple lung cancers. Kyobu Geka.

[6] Mun M, Kohno T (2007). Single-stage surgical treatment of synchronous bilateral multiple lung cancers. Ann Thorac Surg.

[7] Goldstraw P, Chansky K, Crowley J, Rami-Porta R, Asamura H, Eberhardt WE (2016). The IASLC lung Cancer staging project: proposals for revision of the TNM stage groupings in the forthcoming (eighth) edition of the TNM classification for lung Cancer. J Thorac Oncol.

[8] Martini N, Melamed MR (1975). Multiple primary lung cancers. J Thorac Cardiovasc Surg.

[9] Antakli T, Schaefer RF, Rutherford JE, Read RC (1995). Second primary lung cancer. Ann Thorac Surg.

[10] Miskovic A, Lumb AB (2017). Postoperative pulmonary complications. Br J Anaesth.

[11] Romaszko AM, Doboszynska A (2018). Multiple primary lung cancer: a literature review. Adv Clin Exp Med.

[12] Travis WD, Brambilla E, Noguchi M, Nicholson AG, Geisinger KR, Yatabe Y, Beer DG, Powell CA, Riely GJ, van Schil PE, Garg K, Austin JHM, Asamura H, Rusch VW, Hirsch FR, Scagliotti G, Mitsudomi T, Huber RM, Ishikawa Y, Jett J, Sanchez-Cespedes M, Sculier JP, Takahashi T, Tsuboi M, Vansteenkiste J, Wistuba I, Yang PC, Aberle D, Brambilla C, Flieder D, Franklin W, Gazdar A, Gould M, Hasleton P, Henderson D, Johnson B, Johnson D, Kerr K, Kuriyama K, Lee JS, Miller VA, Petersen I, Roggli V, Rosell R, Saijo N, Thunnissen E, Tsao M, Yankelewitz D (2011). International association for the study of lung cancer/american thoracic society/european respiratory society international multidisciplinary classification of lung adenocarcinoma. J Thorac Oncol.

[13] Chen K, Chen W, Cai J, Yang F, Lou F, Wang X, Zhang J, Zhao M, Zhang J, Wang J (2018). Favorable prognosis and high discrepancy of genetic features in surgical patients with multiple primary lung cancers. J Thorac Cardiovasc Surg.

[14] Zhou H, Kang X, Dai L, Yan W, Yang Y, Lin Y, Chen KN (2018). Efficacy of repeated surgery is superior to that of non-surgery for recurrent/second primary lung cancer after initial operation for primary lung cancer. Thoracic Cancer.

[15] Voltolini L, Rapicetta C, Luzzi L, Ghiribelli C, Paladini P, Granato F, Gallazzi M, Gotti G (2010). Surgical treatment of synchronous multiple lung cancer located in a different lobe or lung: high survival in node-negative subgroup. Eur J Cardiothorac Surg.

[16] Ginsberg RJ, Rubinstein LV (1995). Randomized trial of lobectomy versus limited resection for T1 N0 non-small cell lung cancer. Lung Cancer study group. Ann Thorac Surg.

[17] Hattori A, Suzuki K, Takamochi K, Oh S (2015). Is bilateral pulmonary lobectomy feasible in patients with bilateral lung cancers?. Thorac Cardiovasc Surg.

[18] Subramanian M, McMurry T, Meyers BF, Puri V, Kozower BD (2018). Long-term results for clinical stage IA lung Cancer: comparing lobectomy and sublobar resection. Ann Thorac Surg.

[19] Altorki NK, Yip R, Hanaoka T, Bauer T, Aye R, Kohman L, Sheppard B, Thurer R, Andaz S, Smith M, Mayfield W, Grannis F, Korst R, Pass H, Straznicka M, Flores R, Henschke CI, I-ELCAP Investigators (2014). Sublobar resection is equivalent to lobectomy for clinical stage 1A lung cancer in solid nodules. J Thorac Cardiovasc Surg.

[20] Li J, Qiu B, Scarci M, Rocco G, Gao S (2019). Uniportal video-assisted thoracic surgery could reduce postoperative thorax drainage for lung cancer patients. Thoracic Cancer.

[21] Tanvetyanon T, Finley DJ, Fabian T, Riquet M, Voltolini L, Kocaturk C, Bryant A, Robinson L (2015). Prognostic nomogram to predict survival after surgery for synchronous multiple lung cancers in multiple lobes. J Thorac Oncol.

